# New Therapeutics in Promoting and Modulating Mandibular Growth in Cases with Mandibular Hypoplasia

**DOI:** 10.1155/2013/789679

**Published:** 2013-05-29

**Authors:** Tarek El-Bialy, Adel Alhadlaq

**Affiliations:** ^1^7-020D Katz Group Centre for Pharmacy and Health Research, University of Alberta, Edmonton, AB, Canada T6G 2E1; ^2^College of Dentistry, King Saud University, P.O. Box 60169, Riyadh 11545, Saudi Arabia

## Abstract

Children with mandibular growth deficiency may develop airway obstruction. The standard treatment of severe airway obstruction involves invasive procedures such as tracheostomy. Mandibular distraction osteogenesis has been proposed in neonates with mandibular deficiency as a treatment option to avoid tracheostomy procedure later in life. Both tracheostomy and distraction osteogenesis procedures suffer from substantial shortcomings including scarring, unpredictability, and surgical complications. Forward jaw positioning appliances have been also used to enhance mandible growth. However, the effectiveness of these appliances is limited and lacks predictability. Current and future approaches to enhance mandibular growth, both experimental and clinical trials, and their effectiveness are presented and discussed.

## 1. Introduction

 Underdeveloped mandibles can cause severe psychological and functional impact upon the growing child and may be associated with life-threatening complications such as obstructive sleep apnea (OSA) due to severe airway constriction [1, 2]. The prevalence of OSA in children is approximately 3% [3]. Patients with OSA usually have underdeveloped mandible (mandible) [4, 5]. The mortality rate due to OSA has been reported to reach 15% of the affected individuals [6, 7]. This increased mortality is mainly attributed to the retruded position of the mandible which compromises the airway [8]. 

When the airway is compromised because of severe mandible underdevelopment, jaw-positioning appliances [9], nasopharyngeal airway [10], orthodontic plates with velar extension [11], intubation [12], tongue-lip adhesion [13], continuous positive airway pressure (CPAP) [14], tracheostomy [15], mandibular advancement with orthognathic surgery/distraction osteogenesis [16, 17], or an anterior mandibular positioning device is used to manage the airway obstruction [18, 19].

It is to be noted that tracheotomy and mandibular distraction osteogenesis (DO) have major limitations; they are lifesaving and provide substantial lengthening of the mandible quickly. In spite of recent advancements, the tracheostomy procedure remains a treatment with serious and frequent side effects. These side effects include potential tissue traumatization, injury of the laryngeal or tracheal mucosa, and/or other complications (e.g., pneumothorax, hemorrhage, wound complication, tracheal stenosis, and laryngeal stenosis) [15]. To avoid tracheostomies, distraction osteogenesis of the mandible (surgical lengthening of the mandible) has recently been recommended as a viable option for pediatric patients with upper airway obstruction due to mandibular deficiency [16]. This technique has been described as an alternative to tracheostomy in neonates (6 to 26 days of age) to improve airway and breathing [17]. 

Conversely, improving the airway in OSA adult patients with the use of a removable mandibular advancement device (MAD) has recently been shown to have a success rate of only 54.8% [18]. Advancing the mandible with oral appliances depends solely on patient's compliance and has been reported to be effective in short term only [19]. Nonetheless, the long-term efficacy of all above-mentioned treatment modalities is unknown. 

## 2. Mandibular Growth

The condylar cartilage in the mandible is a secondary cartilage. It has been shown that mechanical stimuli are necessary for the normal growth of this type of cartilage [20–24]. Bite-jumping appliances (orthodontic/orthopedic functional appliances) have long been used for “growth modification” of the mandible in the field of orthodontics and craniofacial orthopedics. However, the effectiveness of these appliances has been criticized and is still considered an area of controversy [25]. Other currently available mechanical loading techniques, for example, electroforce 3200 mechanical testing machine, are not clinically applicable for severe mandibular underdevelopment due to the large size of the device (mechanical testing machine) or because patients can not fit within the proposed devices (electroforce or mechanical testing machines) [20]. 

## 3. Current Treatment Modalities and Challenges

### 3.1. Bite-Jumping Appliances (Functional Appliances)

 Growth modification of the mandible using functional appliances (FAs) has been used to enhance forward positioning of the mandible. They are commonly used clinically to enhance mandibular growth in patients with underdeveloped mandibles. Recent animal experiments have demonstrated significant increase in the endochondral ossification (bone formation within the growing cartilage) at the mandibular condyle in response to the mandibular protrusive forces [20, 26, 27]. This forward mandibular positioning has been hypothesized to solicit a sequence of cellular events that lead to increased vascularization, new bone formation, and enhanced condylar growth [28]. Also, a recent clinical trial on the effectiveness of twin block functional appliance on mandibular growth in 15 boys and 19 girls ranging in age from 9 years 3 months to 10 years 8 months at the start of treatment showed that its use increases the mandibular length 2.3 mm more than that of a control group [29]. Controversially, other clinical trials of FAs therapy have demonstrated either no substantial growth enhancement or increased mandibular growth only at the initial stage, with the growth phenotype of the mandible returning to its original pattern afterwards [30, 31]. Interestingly, FAs were reported to increase the number of replicating mesenchymal stem cells in growing rats at both the mandibular condyle and the glenoid fossa [32]. In a related study, a correlation was demonstrated between the application of FAs as a mechanical stimulator and the number of stem cells in mandibular condyles and the glenoid fossa [33]. These studies demonstrated that the number of mesenchymal cells in a given locus normally determines the potential for bone growth in that area and that the number of mesenchymal cells in the glenoid fossa is directly correlated with the amount of bone produced during natural growth and mandibular advancement [32, 33]. It has been hypothesized that a lack of native stem cells in the mandibular condyle and glenoid fossa contributes to the underdevelopment of the mandible [32, 33]. Consequently, development of new techniques to foster stem cell recruitment to the growing condyles and the glenoid fossa becomes practical. 

Although the exact mechanism of FAs involvement in promoting mandibular growth is not fully understood, previous reports have shown that FAs enhance mandibular growth through an increase in the production of Runx2 [27], which is a transcription factor that belongs to the Runt domain gene family. The Runx2 promotes osteoblast's differentiation and function by transcriptionally upregulating all the major osteoblast-specific genes, including osteocalcin, type-I collagen, bone sialoprotein, osteopontin, alkaline phosphatase, and collagenase-3 [34]. Also, FAs enhance Sox9 and type II collagen expression in rats [35], a result that has been confirmed by immunostaining techniques [20, 26–28].

### 3.2. Low-Intensity Pulsed Ultrasound

**Figure 1:**
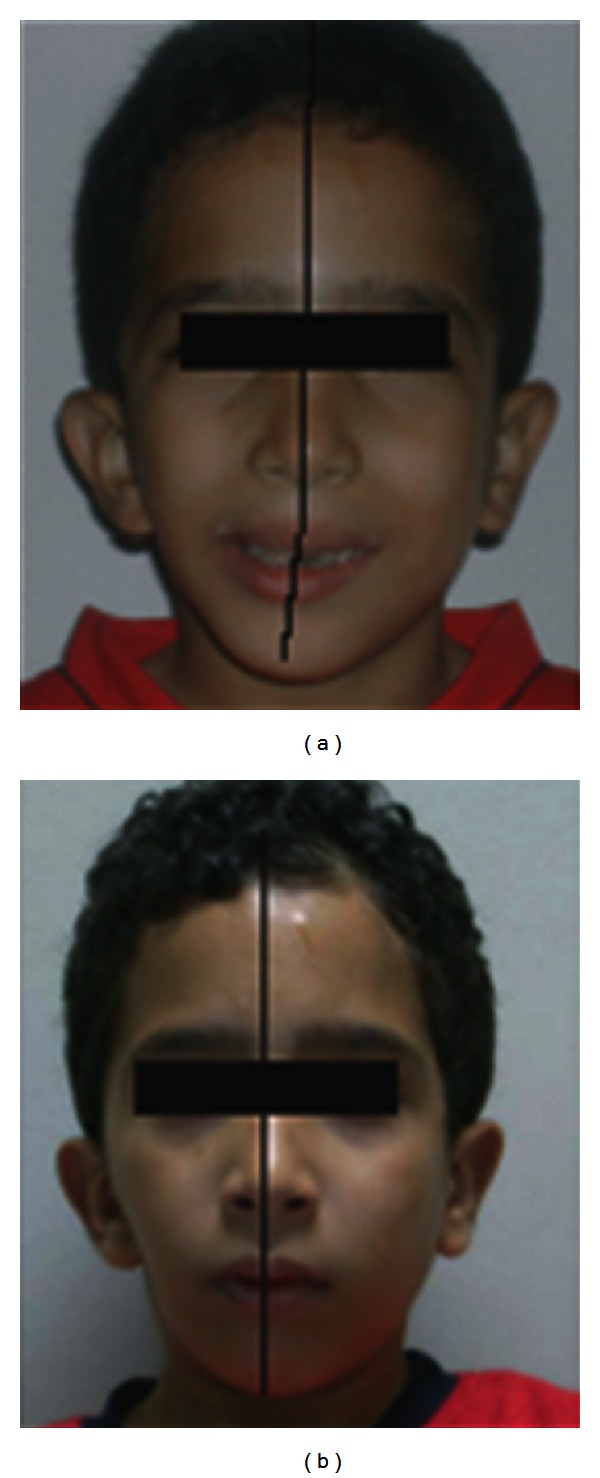
A hemifacial microsomia patient treated with 20 minutes LIPUS per day for a year with a hybrid functional appliance. (a) Before and (b) after treatment.

Low-intensity pulsed ultrasound (LIPUS) produces mechanical waves that propagate through biological tissues at a pulse frequency of 1.5 MHz with a pulse repetition frequency of 1 kHz. At an output power of 30 mW/cm^2^, LIPUS can stimulate tissue growth without heating [15, 16]. A daily treatment with LIPUS for 20 minutes has been established as a favorable treatment modality in the field of orthopedics [36–39]. This treatment protocol has been found to stimulate bone healing after fracture in a variety of human and animal models by promoting new vascularization and bone formation [35–37]. Also, this technique has been applied successfully to promote growth and healing after distraction (i.e., excessive separation of bony segments) of the tibia in a rabbit model and after distraction of the callus [36, 39]. Daily direct application of LIPUS for 4 weeks also stimulated mandibular bone growth in rats and rabbits [22, 23]. However, achieving similar results in monkeys required four months of treatment [40] and about a year in humans (when combined with FAs) [41] (Figure 1). Such long periods of daily application of LIPUS are challenging and highly demanding for a clinical implementation. Thus, the development of an approach to boost the stimulatory effect of LIPUS on bone growth is necessary. 

Recent reports have shown that LIPUS has anabolic effect in chondrocytes with increased stimulation when LIPUS is applied for longer durations [42]. Furthermore, the current consensus in the literature is that the stimulatory effect of therapeutic ultrasound on bone formation is dose dependent (daily treatment time) [42–44]. Angiogenesis has also been shown to be increased with LIPUS treatment, thus improving the blood flow to the treatment area that is critical for bone growth [45, 46]. In addition, it has been shown that LIPUS promotes stem cell expansion and differentiation [47–49]. Indeed, the application of ultrasound in stem cell expansion and differentiation as well as in bone mineralization and regeneration has gained considerable attention in recent years [50, 51].

The mechanism of mandibular growth enhancement by LIPUS is not fully understood. The hypothesized mechanism of action of LIPUS to stimulate mandibular growth is through increase in vascular endothelial growth factor (VEGF) and Runx2 in bone healing since these have been shown to be correlated to increased mandibular growth [46]. LIPUS is known to increase the expression of VEGF and Runx2 in bone healing [45]. Also, LIPUS has been shown to increase the expression of osteocalcin, Runx2, and bone sialoprotein in stem cells [52]. In addition, the expression of Runx2, Msx2, Dlx5, osterix, bone sialoprotein, and bone morphogenetic protein-2 has been shown to be enhanced in MG-63 osteoblasts [53]. Sox9 is known to be a crucial molecule in stem cell proliferation, condensation, and chondrocyte differentiation [54–56]. Runx2 is involved in chondrocyte differentiation, whereas VEGF and collagen type X are involved in endochondral ossification [46, 54]. Integrins may act as mechanotransducers that can transform acoustic pulsed energy into intracellular biochemical signals that subsequently induce cell proliferation [57]. Although the mechanisms are still to be uncovered, current knowledge indicates that FAs and LIPUS have a potential synergistic effect in enhancing mandibular growth through mutual upregulation of Sox9, Runx2, and type-II collagen. This synergistic effect may be further increased by neovascularization produced by local application of bone marrow stem cells to mandibular condyles [58–64]. A critical need still exists for an optimized and effective technique to promote mandibular growth in a reasonable period of time (2-3 months in humans) for clinical application. Yet, it is to be tested the effect of LIPUS on increase of Sox9, Runx2, VEGF, and Type-II collagen in mandibular condyles treated by LIPUS. It is possible that once LIPUS parameters are optimize (Frequency, intensity, and treatment time), they may be used clinically with minimum compliance of growing children with underdeveloped mandibles. 

Growth hormone: growth hormone (GH) is an anterior pituitary hormone that induces general growth including bone [65–69]. It has been reported that systemic administration of GH enhances bone formation in animals [68]. Also, GH plays an important role not only in skeletal growth and development in young people but also in regulating bone remodeling throughout life [70]. Cell surface receptors for GH have been reported to be present in the temporomandibular joint (TMJ) [71]. 

Children undergoing GH therapy for short stature or isolated GH deficiency (who usually have normal jaw size) can experience a burst in jaw growth while on the GH therapy [72, 73].

**Figure 2:**
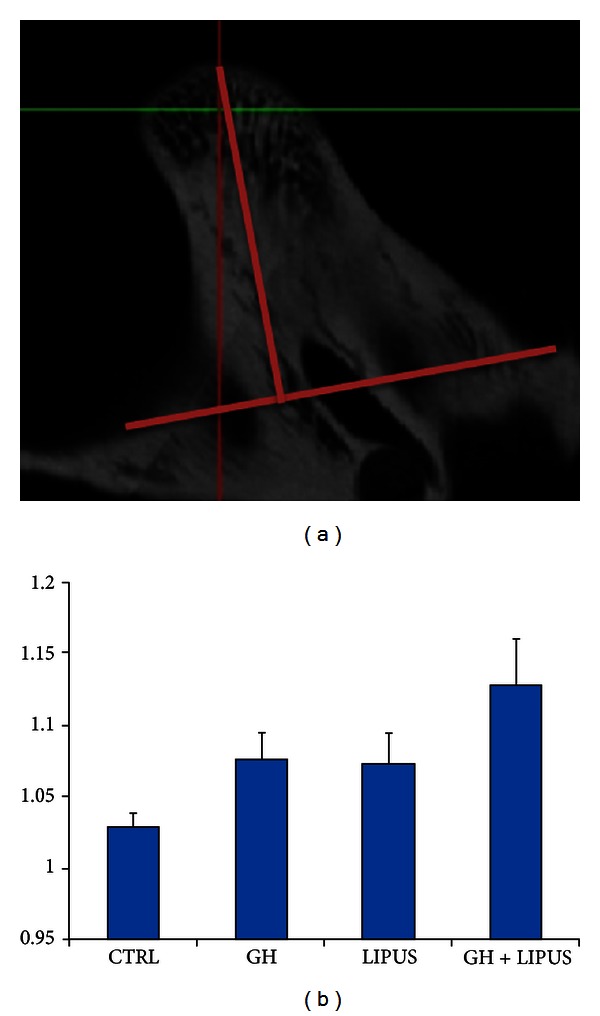
Effect of LIPUS and growth hormone on mandibular head length changes. (a) Measurement of mandibular head. (Straight line was drawn in between the two points that are anatomically situated in the lowest position in 2D reconstructed images. Length of mandibular head was measured by drawing a perpendicular line from the highest point of the condylar process towards the former line.) (b) Effect of treatment on the length of the mandibular head (LMH). After measurement length of the left mandibular head was divided by length of the right mandibular head. Ratio was analyzed among four different groups mentioned before. *P < 0.05, compared to control.

In spite of the potential side effects of GH administration such as inducing body weight gain [74] and upregulation of proto-oncogenes like C-jun in liver [75], kidney, and other vital tissues [76], there has been an attempt to enhance mandibular growth by local injection of recombinant growth hormone (rGH) into the posterior attachment of mandibular condyle of growing rats with or without LIPUS application [77]. The hypothesized mechanism of action of local rGH application is to increase endochondoral bone formation in the mandibular condyles without possible side effect of systemic use of rGH. The findings indicated that local rGH injection into mandibular condyles in rats increased mandibular growth compared to the control group. The study concluded that the used rGH dose does not have synergistic effect in combination with LIPUS application in enhancing mandibular bone volume or mandibular surface area while the combined treatment increased mandibular head length compared to either treatment alone (Figures 2(a) and 2(b)). Also, local injection of rGH increased C-jun in the liver [77]. Therefore, it seems to be prudent to have an optimized rGH/LIPUS protocol to enhance mandibular growth with minimum potential side effects and to study the effect of rGH in the long term. Future studies are needed to test the hypothesis that local injection of different rGH doses to mandibular condyle can modulate molecular mechanisms of mandibular growth, especially Runx2, VEGF, Sox9, and type-II collagen. Until the optimized rGH dose with or without LIPUS application can stimulate mandibular growth with minimum or no systemic side effect, the possible clinical application of rGH may not be foreseen.

### 3.3. Photobiomodulation

Photobiomodulation has been recently reported to be an effective therapeutic technique by which low-level laser light is used to enhance tissue growth and regeneration. This technique has been shown to minimize pain after orthodontic appliance adjustment [78–80] and minimize inflammation caused by the orthodontic appliances [81]. Low-level laser (LLL) therapy is a form of photobiomodulation that has been shown to enhance bone formation in the midpalatal suture after orthodontic maxillary expansion [82]. Also, the use of LLL has been proposed to accelerate tooth movement by inducing alveolar bone remodeling in the form of bone formation and cell proliferation in the tension side as well as increase the number of osteoclasts in the compression side of the orthodontically moved teeth [83, 84]. 

**Figure 3:**
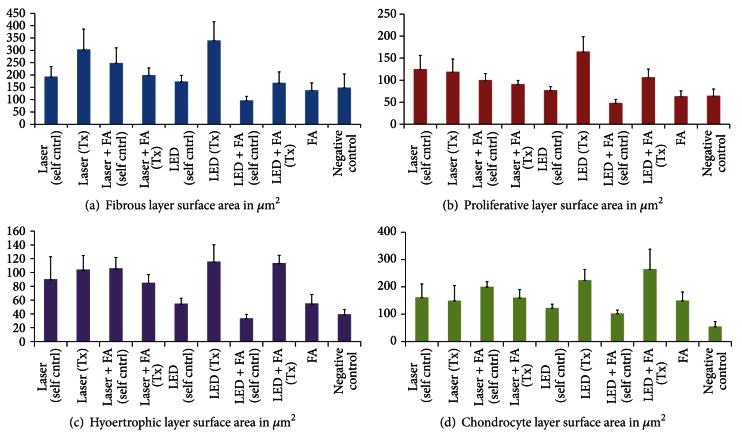
Surface area of mandibular condylar layers. (a) Fibrous, (b) proliferative, (c) hypertrophic, and (d) cartilaginous layers in rats treated by LED, laser with or without functional appliance (FA). It can be seen that LED increases surface areas in (a), (b), and (d) compared to control and almost similar to control in (c).

Light emitting diode (LED) is a monochromatic red-to-near infrared (NIR) light that has been shown to enhance retinal function in an animal model when used in the range of 630–1000 nm [85]. The wavelength of LED is close to that used in LLL (600–1000 nm) with similar energy [85]. Both the LED and the LLL result in photobiomodulation effects [86–88]. However, the difference between LED and the LLL is that LLL is a LASER with the characteristic of coherency, whereas LED light is not coherent, and therefore it is not expected to result in any side effects [86–89]. The LED can also be produced at a lower cost compared to the LLL and it can be safely applied to a larger area of the body surface. It has been shown that LED light can alter the cellular metabolism following absorption by a cellular photoacceptor known as cytochrome c-oxidase [85, 89]. Moreover, it has been hypothesized that LED-mediated photobiomodulation may have potential in stimulating mandibular growth. A pilot study on the effect of LLL and LED on mandibular growth stimulation reported that both LED and LLL can stimulate mandibular growth in growing rats [90]. The objective of this pilot study was to compare the possible effect of LLL and LED of similar wavelength (850 nm) and energy output (6 J/cm^2^) with or without the use of functional appliance (FA) on mandibular condylar growth in growing rats. The experimental design of this pilot study was as follows. Twenty-four growing Sprague-Dawley rats (6 weeks old) were divided into six groups of four animals each. Group 1 received LLL, group 2 received LLL + FA, group 3 received LED, group 4 received LED + FA, group 5 received FA and was used as a positive control group, and group 6 received no treatment and was used as negative control group. Animals were treated for ten minutes with the corresponding treatment every day on the right side of the mandibular condyle for four weeks. The results showed that there is a statistically significant increase (60%) in the FL in the treated condyles by the laser group compared to the counte control condyles. Also, LED increased both the FL and CL significantly and PL more than the negative control condyles (*P* < 0.05). Although LED and FA significantly increased all condylar layers when compared to the untreated negative control condyles, LED alone was more effective in increasing condylar layers than the combined LED and FA treatment (*P* < 0.05). On the other hand, LED and FA increased the CL more than the LLL and FA group (*P* < 0.05). The findings of this study suggest that LED is better than LLL in stimulating mandibular condyles in growing rats (Figure 3) [90]. However, the exact mechanism by which LED or LLL stimulate mandibular growth is yet to be understood fully so that an optimum technique can be developed using photobiomodulation. Future studies are needed to test the hypothesis that LED treatment to mandibular condyle can modulate molecular mechanisms of mandibular growth, especially Runx2, VEGF, Sox9, and type-II collagen.

Photobiomodulation for stimulation of mandibular condylar growth may be close to application in the clinic because clinical trials using LED in patients undergoing orthodontic treatment have identified enhanced tooth movement and minimal side effects, such as root resorption.

### 3.4. Gene Therapy

Gene therapy involves either physical or chemical transfer of genetic material in the host cell, [82]. Gene therapy involves using plasmid DNA alone (naked DNA) [91, 92] or associated with gene carriers or vectors such as nonviral vectors (liposomes or a polymer matrix) [93]. Liposomes have proven sufficient for gene transfer into chondrocytes, and they have several advantages over adenovirus vectors including ease of preparation, lack of limitations on the size of the DNA, and minimum immunological reaction [94]. However, *in vivo* or clinical application is yet to be proven. Viral vectors carrying vascular endothelial growth factor (rAAV-VEGF) have been shown to stimulate mandibular growth *in vivo* in rats [95]. Yet, more research is needed to optimize the technique and detailed toxicity evaluation of viral and nonviral vectors (both local and systemic), and testing optimized techniques in higher animals before clinical trials can be conducted. The hypothesis underlying local injection of vector-loaded VEGF into mandibular condyles is that this VEGF can modulate mandibular growth through added VEGF effect that has been shown to be correlated to mandibular growth stimulation [27, 28]. VEGF may stimulate mandibular growth through two mechanisms: (1) through stimulation of endochondral bone growth and (2) through recruitment of new replicating mesenchymal stem cells which is correlated to mandibular growth [32, 33]. It seems that the road towards clinical application of gene therapy to enhance mandibular condylar growth is long compared to other modalities like LIPUS or LED. Many questions remain to be answered regarding the safety, optimization, and mechanism underlying gene therapy, which is also more invasive and currently less accepted than LIPUS or LED.

## 4. Syndromic Mandibular Hypoplasia and Treatment Possibilities

Syndromic mandibular hypoplasia, as in hemifacial microsomia (HFM), is distinct from symmetrical mandibular hypoplasia. HFM is a congenital anomaly that is presented by asymmetric facial structures in which the mandible and overlying structures fail to develop normally. HFM is also known as otomandibular dysostosis, [96] first and second branchial arch syndrome, [97, 98] oculo-auriculovertebral dysplasia, [99] Goldenhar syndrome, [100, 101] lateral facial dysplasia; [102] and craniofacial microsomia [103, 104]. The prevalence of HFM has been previously reported as 1 in 3000 or 1 in 5600 births [105–108]. Males are more affected than females [109], and it has been reported that the right side is affected more than the left side (3 : 2 ratio) [110]. At present, the underlying cause of HFM remains unknown. It has been hypothesized that HFM results from a developmental abnormality related to hemorrhage and rupture of the stapedial artery (a small blood vessel near the ear), as supported by mouse studies [109–111]. It is to be noted that small animal models of HFM cannot be extrapolated to humans, as there is no published reports that indicate that hypothesized etiology of human HFM is similar to those in lower animal models. Pruzansky classification is the most known HFM classification used by clinicians and researchers [112–114]. Treatment of HFM depends on each case severity and patient's age. Treatment of HFM may include orthodontic hybrid functional appliances in less severe cases and/or surgical intervention utilizing orthognathic surgery or distraction osteogenesis [115]. Since the etiology of HFM is not fully understood, the use of the new proposed techniques may not be fully applicable to syndromic cases like HFM. 

## 5. Current Challenges in Available Techniques Used to Enhance Mandibular Growth

As noted above, although LIPUS, LED, GH, or gene therapy may be future techniques that may be used one day for mandibular stimulation in patients with underdeveloped mandibles, the following challenges are foreseen for these techniques. While optimized LIPUS treatment is hypothesized to be dose (treatment-time) dependent, it is a challenge to use LIPUS daily for more than 20 minutes per day to mandibular condyles, especially in growing children. From the preliminary study that showed a proof of principle that local rGH injection can enhance mandibular growth, possible increase in rGH dose might bring a risk of systemic unwanted effect(s). While LED treatment to growing mandibles show promising effect, the underlying mechanisms that are involved in LED-mediated mandibular growth stimulation are not known; hence, possible optimized technique of LED application is not known or cannot be hypothesized. Finally, it seems to be very early for proposing genetherapy for human mandibular growth due to the following challenges. It is not known whether locally injected vector-loaded genes have possible systemic effect or not. Although nonviral vectors have been investigated for possible future use in humans, it is not known the possible side effects of these nonviral vectors. It is also not known the optimum dose of each vector or vector-loaded gene concentration that can enhance mandibular growth without inducing unnecessary overgrowth of the mandibles or inducing neoplastic growth. With these challenges, future research may be directed towards uncovering these mechanisms and studying possible side effects as well as optimized techniques in mandibular growth stimulation.

Although shown to be a clinically acceptable treatment modality, bite-jumping appliances (functional appliances (Fas)) alone may not be fully effective in stimulating mandibular growth to the level that they can substitute surgical repositioning of the mandible in severe mandibular deficiency cases. LIPUS can stimulate mandibular growth in growing animals and in humans; however, an optimized technique to shorten treatment time requires further investigations in lower and higher animals before any clinical trials may be proposed. An optimized technique that utilizes local rGH administration with or without LIPUS is worth investigation to stimulate mandibular growth with minimum potential side effects. Gene therapy as well as LLL or LED seems to be promising approaches in stimulating mandibular growth. However, detailed toxicity investigations of these techniques are required before potential clinical trials can be performed.

## References

[1] Sunitha C, Kumar SA (2010). Obstructive sleep apnea and its management. *Indian Journal of Dental Research*.

[2] Chang ET, Shiao GM (2008). Craniofacial abnormalities in Chinese patients with obstructive and positional sleep apnea. *Sleep Medicine*.

[3] Chang SJ, Chae KY (2010). Obstructive sleep apnea syndrome in children: epidemiology, pathophysiology, diagnosis and sequelae. *Korean Journal of Pediatrics*.

[4] Higurashi N, Kikuchi M, Miyazaki S, Itasaka Y (2001). Comparison of Ricketts analysis and Downs-Northwestern analysis for the evaluation of obstructive sleep apnea cephalograms. *Psychiatry and Clinical Neurosciences*.

[5] Vieira BB, Itikawa CE, de Almeida LA (2011). Cephalometric evaluation of facial pattern and hyoid bone position in children with obstructive sleep apnea syndrome. *International Journal of Pediatric Otorhinolaryngology*.

[6] Lavie P, Herer P, Peled R (1995). Mortality in sleep apnea patients: a multivariate analysis of risk factors. *Sleep*.

[7] Lavie P, Herer P, Lavie L (2007). Mortality risk factors in sleep apnoea: a matched case-control study. *Journal of Sleep Research*.

[8] Cohen RB (1995). Obstructive sleep apnea: a mandibular positioning device for treatment and diagnosis of an obstruction site. *Compendium of Continuing Education in Dentistry*.

[9] Villa MP, Bernkopf E, Pagani J, Broia V, Montesano M, Ronchetti R (2002). Randomized controlled study of an oral jaw-positioning appliance for the treatment of obstructive sleep apnea in children with malocclusion. *American Journal of Respiratory and Critical Care Medicine*.

[10] Huo H, Li WY, Shen P, Liu JH (2010). One night treatment of obstructive sleep apnea and hypopnea syndrome with nasopharyngeal airway. *Zhonghua Er Bi Yan Hou Tou Jing Wai Ke Za Zhi*.

[11] Hochban W, Hoch B (1998). Obstructive sleep apnoea in children: an interdisciplinary approach with special regard to craniofacial disorders. *Pneumologie*.

[12] Corso RM, Piraccini E, Calli M (2011). Obstructive sleep apnea is a risk factor for difficult endotracheal intubation. *Minerva Anestesiologica*.

[13] Freed G, Pearlman MA, Brown AS, Barot LR (1988). Polysomnographic indications for surgical intervention in Pierre Robin sequence: acute airway management and follow-up studies after repair and take-down of tongue-lip adhesion. *Cleft Palate Journal*.

[14] Waters KA, Everett FM, Bruderer JW, Sullivan CE (1995). Obstructive sleep apnea: the use of nasal CPAP in 80 children. *American Journal of Respiratory and Critical Care Medicine*.

[15] Kremer B, Botos-Kremer AI, Eckel HE, Schlöndorff G (2002). Indications, complications, and surgical techniques for pediatric tracheostomies—an update. *Journal of Pediatric Surgery*.

[16] Miloro M (2010). Mandibular distraction osteogenesis for pediatric airway management. *Journal of Oral and Maxillofacial Surgery*.

[17] Denny A, Kalantarian B (2002). Mandibular distraction in neonates: a strategy to avoid tracheostomy. *Plastic and Reconstructive Surgery*.

[18] Tison C, Sébille-Elhage S, Ferri J (2011). Mandibular advancement device: a 5-year long experience in obstructive sleep apnea/hypopnea syndrome. *Revue de Stomatologie et de Chirurgie Maxillo-Faciale*.

[19] Lam B, Sam K, Lam JCM, Lai AYK, Lam CL, Ip MSM (2011). The efficacy of oral appliances in the treatment of severe obstructive sleep apnea. *Sleep and Breathing*.

[20] Sobue T, Yeh WC, Chhibber A (2011). Murine TMJ loading causes increased proliferation and chondrocyte maturation. *Journal of Dental Research*.

[21] Rabie AB, Hägg U (2002). Factors regulating mandibular condylar growth. *American Journal of Orthodontics and Dentofacial Orthopedics*.

[22] Oyonarte R, Zárate M, Rodriguez F (2009). Low-intensity pulsed ultrasound stimulation of condylar growth in rats. *Angle Orthodontist*.

[23] El-Bialy T, El-Shamy I, Graber TM (2003). Growth modification of the rabbit mandible using therapeutic ultrasound: is it possible to enhance functional appliance results?. *Angle Orthodontist*.

[24] Peltomäki T, Kylämarkula S, Vinkka-Puhakka H, Rintala M, Kantomaa T, Rönning O (1997). Tissue-separating capacity of growth cartilages. *European Journal of Orthodontics*.

[25] Shen G, Darendeliler MA (2006). Cephalometric evaluation of condylar and mandibular growth modification: a review. *Orthodontics & Craniofacial Research*.

[26] Shen G, Rabie AB, Hägg U, Chen RJ (2003). Neovascularization in condylar cartilage in response to mandibular protrusion. *Chinese Journal of Dental Research*.

[27] Rabie ABM, Shum L, Chayanupatkul A (2002). VEGF and bone formation in the glenoid fossa during forward mandibular positioning. *American Journal of Orthodontics and Dentofacial Orthopedics*.

[28] Rabie ABM, Leung FYC, Chayanupatkul A, Hägg U (2002). The correlation between neovascularization and bone formation in the condyle during forward mandibular positioning. *Angle Orthodontist*.

[29] Sidlauskas A (2005). Clinical effectiveness of the Twin block appliance in the treatment of Class II Division 1 malocclusion. *Stomatologija*.

[30] Du X, Hägg U, Rabie ABM (2002). Effects of headgear Herbst and mandibular step-by-step advancement versus conventional Herbst appliance and maximal jumping of the mandible. *European Journal of Orthodontics*.

[31] Bendeus M, Hägg U, Rabie B (2002). Growth and treatment changes in patients treated with a headgear-activator appliance. *American Journal of Orthodontics and Dentofacial Orthopedics*.

[32] Rabie ABM, Wong L, Tsai M (2003). Replicating mesenchymal cells in the condyle and the glenoid fossa during mandibular forward positioning. *American Journal of Orthodontics and Dentofacial Orthopedics*.

[33] Rabie ABM, Wong L, Hägg U (2003). Correlation of replicating cells and osteogenesis in the glenoid fossa during stepwise advancement. *American Journal of Orthodontics and Dentofacial Orthopedics*.

[34] Tang GH, Rabie ABM (2005). Runx2 regulates endochondral ossification in condyle during mandibular advancement. *Journal of Dental Research*.

[35] Rabie ABM, She TT, Harley VR (2003). Forward mandibular positioning up-regulates SOX9 and type II collagen expression in the glenoid fossa. *Journal of Dental Research*.

[36] Heckman JD, Ryaby JP, McCabe J, Frey JJ, Kilcoyne RF (1994). Acceleration of tibial fracture-healing by non-invasive, low-intensity pulsed ultrasound. *Journal of Bone and Joint Surgery. American*.

[37] Kristiansen TK, Ryaby JP, McCabe J, Frey JJ, Roe LR (1997). Accelerated healing of distal radial fractures with the use of specific, low-intensity ultrasound: a multicenter, prospective, randomized, double- blind, placebo-controlled study. *Journal of Bone and Joint Surgery. American*.

[38] Shimazaki A, Inui K, Azuma Y, Nishimura N, Yamano Y (2000). Low-intensity pulsed ultrasound accelerates bone maturation in distraction osteogenesis in rabbits. *Journal of Bone and Joint Surgery. British*.

[39] Mayr E, Laule A, Suger G, Rüter A, Claes L (2001). Radiographic results of callus distraction aided by pulsed low-intensity ultrasound. *Journal of Orthopaedic Trauma*.

[40] El-Bialy T, Hassan A, Albaghdadi T, Fouad HA, Maimani AR (2006). Growth modification of the mandible with ultrasound in baboons: a preliminary report. *American Journal of Orthodontics and Dentofacial Orthopedics*.

[41] El-Bialy T, Hasan A, Alyamani A, Albaghdadi T (2010). Treatment of hemifacial microsomia by therapeutic ultrasound and hybrid functional appliance. A non-surgical approach. *Open Access Journal of Clinical Trials*.

[42] Schumann D, Kujat R, Zellner J (2006). Treatment of human mesenchymal stem cells with pulsed low intensity ultrasound enhances the chondrogenic phenotype in vitro. *Biorheology*.

[43] El-Bialy T, Royston TJ, Magin RL, Evans CA, Zaki AEM, Frizzell LA (2002). The effect of pulsed ultrasound on mandibular distraction. *Annals of Biomedical Engineering*.

[44] Chan CW, Qin L, Lee KM, Cheung WH, Cheng JCY, Leung KS (2006). Dose-dependent effect of low-intensity pulsed ultrasound on callus formation during rapid distraction osteogenesis. *Journal of Orthopaedic Research*.

[45] Young SR, Dyson M (1990). The effect of therapeutic ultrasound on angiogenesis. *Ultrasound in Medicine and Biology*.

[46] Dai J, Rabie ABM (2007). VEGF: an essential mediator of both angiogenesis and endochondral ossification. *Journal of Dental Research*.

[47] Ang WT, Scurtescu C, Hoy W, El-Bialy T, Tsui YY, Chen J (2010). Design and implementation of therapeutic ultrasound generating circuit for dental tissue formation and tooth-root healing. *IEEE Transactions on Biomedical Circuits and Systems*.

[48] Aldosary TA, Uludag H, Doschak M, Chen J, Tsui Y, EL-Bialy T Effect of ultrasound on human umbilical cord perivascular-stem cell expansion.

[49] Marvel S, Okrasinski S, Bernacki SH, Loboa E, Dayton PA (2010). The development and validation of a lipus system with preliminary observations of ultrasonic effects on human adult stem cells. *IEEE Transactions on Ultrasonics, Ferroelectrics, and Frequency Control*.

[50] Suzuki A, Takayama T, Suzuki N, Sato M, Fukuda T, Ito K (2009). Daily low-intensity pulsed ultrasound-mediated osteogenic differentiation in rat osteoblasts. *Acta Biochimica et Biophysica Sinica*.

[51] Nishizawa K, Imai S, Mimura T (2010). In-advance trans-medullary stimulation of bone marrow enhances spontaneous repair of full-thickness articular cartilage defects in rabbits. *Cell and Tissue Research*.

[52] Jiang T, Xu T, Gu F, Chen A, Xiao Z, Zhang D (2012). Osteogenic effect of low intensity pulsed ultrasound on rat adipose-derived stem cells in vitro. *Journal of Huazhong University of Science and Technology. Medical sciences*.

[53] Leskinen JJ, Karjalainen HM, Olkku A, Hynynen K, Mahonen A, Lammi MJ (2008). Genome-wide microarray analysis of MG-63 osteoblastic cells exposed to ultrasound. *Biorheology*.

[54] Las Heras F, Gahunia HK, Pritzker KP (2012). Articular cartilage development: a molecular perspective. *Orthopedic Clinics of North America*.

[55] Mostafa NZ, Uludağ H, Dederich DN, Doschak MR, El-Bialy T (2009). Anabolic effects of low-intensity pulsed ultrasound on human gingival fibroblasts. *Archives of Oral Biology*.

[56] El-Bialy T, Uludag H, Jomha N, Badylak SF (2010). In vivo ultrasound-assisted tissue-engineered mandibular condyle: a pilot study in rabbits. *Tissue Engineering, Part C*.

[57] Zhou S, Schmelz A, Seufferlein T, Li Y, Zhao J, Bachem MG (2004). Molecular mechanisms of low intensity pulsed ultrasound in human skin fibroblasts. *Journal of Biological Chemistry*.

[58] Matsumoto T, Cooper GM, Gharaibeh B (2009). Cartilage repair in a rat model of osteoarthritis through intraarticular transplantation of muscle-derived stem cells expressing bone morphogenetic protein 4 and soluble Flt-1. *Arthritis and Rheumatism*.

[59] Pei M, He F, Boyce BM, Kish VL (2009). Repair of full-thickness femoral condyle cartilage defects using allogeneic synovial cell-engineered tissue constructs. *Osteoarthritis and Cartilage*.

[60] Grigolo B, Lisignoli G, Desando G (2009). Osteoarthritis treated with mesenchymal stem cells on Hyaluronan-based scaffold in rabbit. *Tissue Engineering, Part C*.

[61] Toghraie FS, Chenari N, Gholipour MA (2011). Treatment of osteoarthritis with infrapatellar fat pad derived mesenchymal stem cells in Rabbit. *Knee*.

[62] Murphy JM, Fink DJ, Hunziker EB, Barry FP (2003). Stem cell therapy in a caprine model of osteoarthritis. *Arthritis and Rheumatism*.

[63] Hamou C, Callaghan MJ, Thangarajah H (2009). Mesenchymal stem cells can participate in ischemic neovascularization. *Plastic and Reconstructive Surgery*.

[64] Chen K, Man C, Zhang B, Hu J, Zhu SS (2012). Effect of in vitro chondrogenic differentiation of autologous mesenchymal stem cells on cartilage and subchondral cancellous bone repair in osteoarthritis of temporomandibular joint. *International Journal of Oral and Maxillofacial Surgery*.

[65] Ohlsson C, Bengtsson BÅ, Isaksson OGP, Andreassen TT, Slootweg MC (1998). Growth hormone and bone. *Endocrine Reviews*.

[66] Giustina A, Mazziotti G, Canalis E (2008). Growth hormone, insulin-like growth factors, and the skeleton. *Endocrine Reviews*.

[67] Isaksson OGP, Jansson JO, Gause IAM (1982). Growth hormone stimulates longitudinal bone growth directly. *Science*.

[68] Hedner E, Linde A, Nilsson A (1996). Systemically and locally administered growth hormone stimulates bone healing in combination with osteopromotive membranes: an experimental study in rats. *Journal of Bone and Mineral Research*.

[69] Johannsson G, Rosén T, Bosaeus I, Sjöström L, Bengtsson BÅ (1996). Two years of growth hormone (GH) treatment increases bone mineral content and density in hypopituitary patients with adult-onset GH deficiency. *Journal of Clinical Endocrinology and Metabolism*.

[70] Parfitt AM (1991). Growth hormone and adult bone remodeling. *Clinical Endocrinology*.

[71] Visnapuu V, Peltomäki T, Rönning O, Vahlberg T, Helenius H (2001). Growth hormone and insulin-like growth factor I receptors in the temporomandibular joint of the rat. *Journal of Dental Research*.

[72] van Erum R, Mulier M, Cards C, Verbeke G, de Zegher F (1997). Craniofacial growth in short children born small for gestational age: effect of growth hormone treatment. *Journal of Dental Research*.

[73] Forsberg CM, Krekmanova L, Dahllöf G (2002). The effect of growth hormone therapy on mandibular and cranial base development in children treated with total body irradiation. *European Journal of Orthodontics*.

[74] Farris GM, Miller GK, Wollenberg GK, Molon-Noblot S, Chan C, Prahalada S (2007). Recombinant rat and mouse growth hormones: risk assessment of carcinogenic potential in 2-year bioassays in rats and mice. *Toxicological Sciences*.

[75] Murakami Y, Satake M, Yamaguchi-Iwai Y, Sakai M, Muramatsu M, Ito Y (1991). The nuclear protooncogenes c-jun and c-fos as regulators of DNA replication. *Proceedings of the National Academy of Sciences of the United States of America*.

[76] Rotwein P, Granowski AM, Thomas MJ (1994). Rapid nuclear actions of growth hormone. *Hormone Research*.

[77] Khan I, EL-Kadi A, El-Bialy T Effects of growth hormone and ultrasound on mandibular growth in rats: microCT and toxicity analyses.

[78] Lim HM, Lew KKK, Tay DKL (1995). A clinical investigation of the efficacy of low level laser therapy in reducing orthodontic postadjustment pain. *American Journal of Orthodontics and Dentofacial Orthopedics*.

[79] Turhani D, Scheriau M, Kapral D, Benesch T, Jonke E, Bantleon HP (2006). Pain relief by single low-level laser irradiation in orthodontic patients undergoing fixed appliance therapy. *American Journal of Orthodontics and Dentofacial Orthopedics*.

[80] Fujiyama K, Deguchi T, Murakami T, Fujii A, Kushima K, Takano-Yamamoto T (2008). Clinical effect of CO_2_ laser in reducing pain in orthodontics. *Angle Orthodontist*.

[81] Rodrigues MTJ, Ribeiro MS, Grtoth EB, Zezell DM (2002). Evaluation of effects of laser therapy on oral ulceration induced by fixed orthodontic appliances. *Lasers in Surgery and Medicine*.

[82] Saito S, Shimizu N (1997). Stimulatory effects of low-power laser irradiation on bone regeneration in midpalatal suture during expansion in the rat. *American Journal of Orthodontics and Dentofacial Orthopedics*.

[83] Kawasaki K, Shimizu N (2000). Effects of low-energy laser irradiation on bone remodelling during experimental tooth movement in rats. *Lasers in Surgery and Medicine*.

[84] Sun X, Zhu X, Xu C, Ye N, Zhu H (2001). Effects of low energy laser on tooth movement and remodeling of alveolar bone in rabbits. *Hua Xi Kou Qiang Yi Xue Za Zhi*.

[85] Karu TI, Pyatibrat LV, Kolyakov SF, Afanasyeva NI (2005). Absorption measurements of a cell monolayer relevant to phototherapy: reduction of cytochrome c oxidase under near IR radiation. *Journal of Photochemistry and Photobiology B*.

[86] Khadra M, Rønold HJ, Lyngstadaas SP, Ellingsen JE, Haanæs HR (2004). Low-level laser therapy stimulates bone-implant interaction: an experimental study in rabbits. *Clinical Oral Implants Research*.

[87] Eells JT, Wong-Riley MTT, VerHoeve J (2004). Mitochondrial signal transduction in accelerated wound and retinal healing by near-infrared light therapy. *Mitochondrion*.

[88] Whelan HT, Smits RL, Buchman EV (2001). Effect of NASA light-emitting diode irradiation on wound healing. *Journal of Clinical Laser Medicine and Surgery*.

[89] Wong-Riley MTT, Liang HL, Eells JT (2005). Photobiomodulation directly benefits primary neurons functionally inactivated by toxins: role of cytochrome c oxidase. *Journal of Biological Chemistry*.

[90] Ebrahim A, Yeung J, Habib A, EL-Bialy T, AL-Qahtani JM Histomorphometric analysis: effect of laser and LED on mandibular growth.

[91] Wolff JA, Malone RW, Williams P (1990). Direct gene transfer into mouse muscle in vivo. *Science*.

[92] Herweijer H, Wolff JA (2003). Progress and prospects: naked DNA gene transfer and therapy. *Gene Therapy*.

[93] Niidome T, Huang L (2002). Gene therapy progress and prospects: nonviral vectors. *Gene Therapy*.

[94] Park J, Ries J, Gelse K (2003). Bone regeneration in critical size defects by cell-mediated BMP-2 gene transfer: a comparison of adenoviral vectors and liposomes. *Gene Therapy*.

[95] Dai J, Rabie ABM (2008). Gene therapy to enhance condylar growth using rAAV-VEGF. *Angle Orthodontist*.

[96] Francois JJ, Haustrate L (1954). Anomalies colobomateuses du globe oculaire et syndrome du premier arc. *Annales d'Oculistique*.

[97] Stark RB, Saunders DE (1962). The first branchial syndrome. The oral-mandibular-auricular syndrome. *Plastic and Reconstructive Surgery*.

[98] Grabb WC (1965). The first and second branchial arch syndrome. *Plastic and Reconstructive Surgery*.

[99] Gorlin RJ, Jue KL, Jacobsen U, Goldschmidt E (1963). Oculoauriculovertebral dysplasia. *The Journal of Pediatrics*.

[100] Goldenhar M (1952). Association malformatives de l’oeil et de l’oreille, en particulier le syndrome dermoide epibulbaire-appendices auriculaires-fistula auris congenita et ses relations avec la dysotose mandibulo-faciale. *Journal of Human Genetics*.

[101] Gorlin RJ, Pindborg JJ, Cohen MM (1976). *Syndromes of the Head and Neck*.

[102] Ross RB (1975). Lateral facial dysplasia. (First and second branchial arch syndrome, hemifacial microsomia). *Birth Defects: Original Article Series*.

[103] Converse JM, Coccardo PJ, Becker MH, Wood-Smith D, Converse JM, McCarthy JG, Wood-Smith D (1979). Clinical aspects of craniofacial microsomia. *Symposium on Diagnosis and Treatment of Craniofacial Anomalies*.

[104] Horgan JE, Padwa BL, LaBrie RA, Mulliken JB (1995). OMENS-Plus: analysis of craniofacial and extracraniofacial anomalies in hemifacial microsomia. *The Cleft Palate-Craniofacial Journal*.

[105] Cohen MM (1995). Perspectives on craniofacial asymmetry. I. The biology of asymmetry. *International Journal of Oral and Maxillofacial Surgery*.

[106] Cohen MM (1995). Perspectives on craniofacial asymmetry. II. Asymmetric embryopathies. *International Journal of Oral and Maxillofacial Surgery*.

[107] Cohen MM (1995). Perspectives on craniofacial asymmetry. III. Common and/or well-known causes of asymmetry. *International Journal of Oral and Maxillofacial Surgery*.

[108] Cohen MM (1995). Perspectives on craniofacial asymmetry. IV. Hemi-asymmetries. *International Journal of Oral and Maxillofacial Surgery*.

[109] Cousley RRJ, Wilson DJ (1992). Hemifacial microsomia: developmental consequence of perturbation of the auriculofacial cartilage model?. *American Journal of Medical Genetics*.

[110] Wang RR, Andres CJ (1999). Hemifacial microsomia and treatment options for auricular replacement: a review of the literature. *The Journal of Prosthetic Dentistry*.

[111] Robinson LK, Hoyme HE, Edwards DK, Jones KL (1987). Vascular pathogenesis of unilateral craniofacial defects. *Journal of Pediatrics*.

[112] Pruzansky S (1969). Not all dwarfed mandibles are alike. *Birth Defects: Original Article Series*.

[113] Murray LT, Murray JE, Whitaker LA, Randall P (1978). Asymmetries of the lower part of the face. *Symposium on Reconstruction of Jaw Deformities*.

[114] Kaban LB, Mulliken JB, Murray JE (1981). Three-dimensional approach to analysis and treatment of hemifacial microsomia. *Cleft Palate Journal*.

[115] Moulin-Romsée C, Verdonck A, Schoenaers J, Carels C (2004). Treatment of hemifacial microsomia in a growing child: the importance of co-operation between the orthodontist and the maxillofacial surgeon. *Journal of Orthodontics*.

